# Does Motivation for Exercise Influence Post-Exercise Snacking Behavior?

**DOI:** 10.3390/nu7064804

**Published:** 2015-06-15

**Authors:** James A. Dimmock, Kym J. Guelfi, Jessica S. West, Tasmiah Masih, Ben Jackson

**Affiliations:** The University of Western Australia, 35 Stirling Highway, Crawley, Perth, Western Australia 6009, Australia; E-Mails: kym.guelfi@uwa.edu.au (K.J.G.); jessica.west@research.uwa.edu.au (J.S.W.); tasmiah.masih@research.uwa.edu.au (T.M.); ben.jackson@uwa.edu.au (B.J.)

**Keywords:** unhealthy snacking, motivation, exercise, ego depletion, compensation, physiology

## Abstract

It is well established that regular exercise plays an important role in achieving a number of health and wellbeing outcomes. However, certain post-exercise behaviors, including the consumption of unhealthy high-calorie foods, can counteract some of the benefits of physical activity. There are at least three overlapping pathways through which exercise may increase the likelihood of consuming pleasurable but unhealthy foods: through impulsive cognitive processes, reflective cognitive processes, and/or physiological responses. It is argued in this paper that motivation toward exercise can influence each of these pathways. Drawing from literature from various domains, we postulate that controlled exercise motivation, as opposed to autonomous exercise motivation, is more likely to influence each of these pathways in a manner that leaves individuals susceptible to the post-exercise consumption of pleasurable but unhealthy foods.

## 1. Introduction

**Figure 1:**
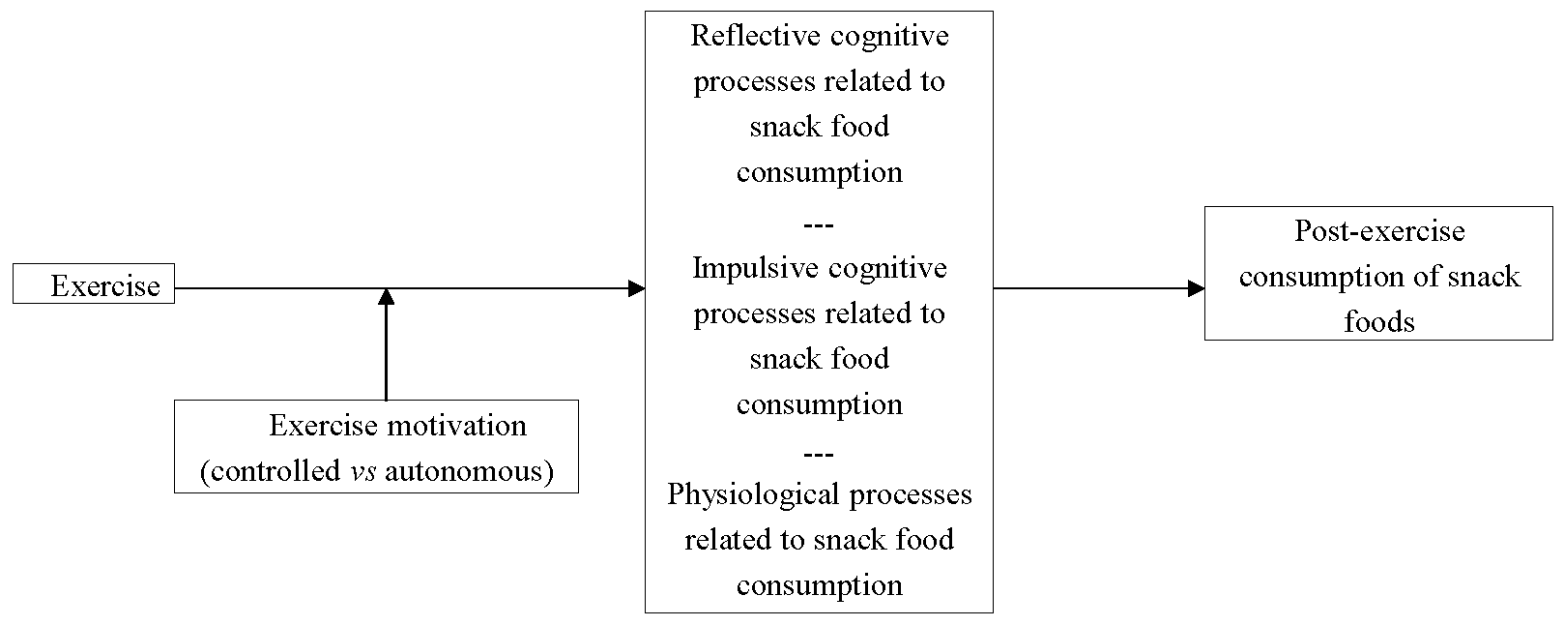
Hypothesized relationships between exercise, exercise motivation, and post-exercise consumption of pleasurable but unhealthy foods.

Energy intake following exercise is extremely variable across individuals and situations (see e.g., [1,2]). Conjecture remains as to the causes of this variability, but evidence is accumulating to indicate that psychological factors associated with both food consumption and exercise are influential in this regard (e.g., [3,4]). In this article, we argue that exercise motivation is likely to influence post-exercise snack consumption (we use a variety of phrases throughout this article in relation to post-exercise food consumption (e.g., “snack consumption”, “snacking”). In all instances though, our focus is on the consumption of hedonically pleasurable but unhealthy foods. We do not wish to offer examples of these foods because there is likely to be both intra- and inter-individual variability in the experience of pleasure during the consumption of specific foods.), and we draw on conceptual and empirical work on self-determination theory [5] to support our argument. First, we overview the nature of exercise motivation from the perspective of self-determination theory, and we also highlight known outcomes of regulations discussed within this theory. Included within this discussion is commentary on two recent studies that support our premise that controlled, as opposed to autonomous, exercise motivation is linked to post-exercise snacking. Our attention then turns to the “why” question—Why is it that this relationship should exist? In addressing this issue, we argue that controlled exercise motivation affects cognitive (both impulsive and reflective) and physiological pathways that frequently precede the consumption of hedonically pleasurable foods. Figure 1 displays the nature of the relationships that are postulated in this article. We hope that our discussion stimulates research on post-exercise snacking, but we also hope to make a broader point that our understanding of health behavior is limited when we consider a given behavior in isolation. More specifically, we feel that a richer understanding of the health consequences of any behavior is obtained when considering how that behavior influences, and is influenced by, the multitude of other behaviors upon which our health is dependent. For now though, our focus is on the effect of exercise motivation on post-exercise snacking, and our discussion begins with a brief overview of motivation as it is described in self-determination theory.

### Exercise Motivation from the Lens of Self-Determination Theory

**Figure 2:**
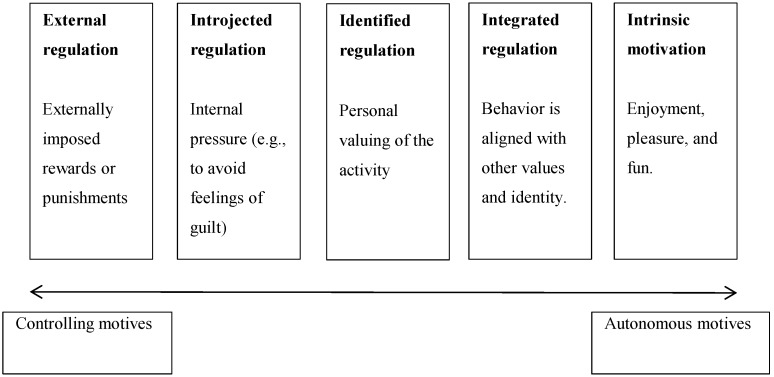
Motivations discussed in self-determination theory and their position in an autonomy continuum.

Physical exercise is a salubrious behavior that is often associated with a variety of positive experiences and outcomes (see [6]). The extent to which these experiences and outcomes are fully understood and accepted, however, varies between people. Some individuals undertake exercise because they fully endorse the instrumental outcomes of the activity (*i.e.*, “identified regulation”), because it is aligned with their values and identity (*i.e.*, “integrated regulation”), and/or because they enjoy the process of exercising (*i.e.*, “intrinsic motivation”). According to proponents of self-determination theory [5], individuals who predominantly cite these reasons for their exercise are said to be *autonomously motivated*. Their motivation comes from a sense of endorsement and is experienced as volitional. Others exercise to obtain externally imposed rewards or avoid externally imposed punishments (*i.e.*, “external regulation”), and/or because they pressure themselves to participate in the activity (*i.e.*, “introjected regulation”). Individuals who mainly cite these reasons to exercise exhibit *controlled motivation*, because their exercise activity is governed by internally or externally imposed pressures. Self-determination theorists sometimes discuss motivation at a level of discrete regulations (e.g., intrinsic motivation), whereas at other times, scholars take a broader perspective, examining the overall extent to which individuals feel self-endorsement and ownership *vs.* pressure over their behavior. We utilize both approaches in this article. That is, our argument occasionally centers on exemplar forms of autonomous or controlled motivation, and on other occasions centers on individuals’ general sense of self-agency in relation to exercise. Figure 2 displays motivations discussed within self-determination as well as their position in an autonomy continuum.

In relation to self-determined motivation in the context of exercise, research has shown that those who cite more autonomous (versus controlled) reasons to exercise, among other things, report fewer cases of exercise relapse [7], report greater exercise intentions [7], exercise more frequently (for review, see [8]), and derive a higher sense of well-being from their exercise (e.g., [9]). Aside from this research, recent work has also focused on the influence of these exercise motivations on post-exercise food consumption. For instance, Werle, Wansink, and Payne [4] found that participants were more prone to seek snacks when reading about “tiring” physical activity as opposed to “fun” physical activity. The concept of “fun” is directly associated with intrinsic motivation, which is the most autonomous form of motivation [5], and although “tiring” is not inherently associated with controlled motivation, past research indicates that it is more likely to be aligned with this type of motivation than with autonomous motivation (see e.g., [10,11]). Other work by Fenzl, Bartsch, and Koenigstorfer [12] has provided even stronger support for the notion that exercise motivation moderates post-exercise snacking. In their study, Fenzl *et al.* [12] invited participants with varying types of exercise motivation to complete a 20 min cycle session labeled as either “fat-burning” or “endurance” training, and then offered participants unhealthy snacks (a large bowl of pretzels). The researchers found an interaction effect between exercise framing and contextual motivation, but in general, autonomously motivated exercisers consumed less of the unhealthy snack food than those exhibiting controlled motivation. The significant interaction effect in this study is certainly worthy of more focus in future work, but the main effect involving motivation is most interesting from our standpoint. The authors built their hypothesis for the role of exercise motivation on post-exercise snacking on the basis of reflective justifications. That is, they speculated that individuals who “self-imposed” physical activity (which was operationalized as controlled motivation) would be more likely to license themselves to food reward, at least under some conditions. They did not assess this mechanism though, and we suggest that this conscious licensing represents only one of at least three pathways that can explain the link between controlled exercise motivation and post-exercise snacking.

## 2. Mechanisms for the Influence of Exercise Motivation on Post-Exercise Snacking

Before we begin our discussion of how exercise motivation might influence conscious licensing (*i.e.*, the mechanism outlined by Fenzl and colleagues [12]), it is important to highlight the difference between this form of cognitive operation and another form that is also key to understanding post-exercise snacking. An assumption in most dominant theories of health behavior (e.g., [13,14]) is that behavior is governed by conscious and deliberate cognitive processing (*i.e.*, conscious reflection, such as licensing). However, an abundance of recent evidence indicates that conscious thought is not always a good predictor of behavior (e.g., [15]), and in light of this issue, dual-process accounts of health behavior have become increasingly popular. These dual-process models describe two broad forms of cognitive processes—Those that are relatively conscious, effortful, slow, and operate efficiently under moderate levels of physiological arousal (herein termed “reflective”), and those that are effortless, fast, and are especially influential under high- or low-levels of physiological arousal (herein termed “impulsive”). Conceivably, these cognitive processes can operate in three different ways to predict behavior: (a) in an additive manner, in which the two types of processes explain a different portion of the variance in the same given behavior; (b) a double dissociation operation, in which the impulsive process predicts spontaneous behavior whereas the reflective process predicts deliberative behavior; and (c) in a multiplicative fashion, in which the impulsive and reflective processes interact to influence behavior [16]. Evidence has emerged to indicate that the double dissociation model is particularly relevant for understanding snacking behavior [16,17,18]. More specifically, research on these dual processes has indicated that reflective processes are often a better predictor of deliberative snacking, whereas impulsive processes are a stronger predictor of spontaneous snacking. The former process will be discussed first; our discussion of the latter process will follow in a subsequent (second) section. In a third section, we discuss the implications of exercise motivation on physiological pathways that are influential in snacking responses.

### 2.1. Exercise Motivation and Reflective Justifications for Snacking

Many individuals have difficulty maintaining an equilibrium between fulfilling immediate desires (e.g., consuming pleasurable snacks) and satisfying long-term goals (e.g., for health) [19,20]. Recently, it has been proposed that one cognitive strategy people employ to reach this equilibrium is to activate compensatory beliefs [21]. These compensatory beliefs reflect the idea that the negative effects of one behavior can be neutralized or compensated for by the positive effects of another. According to the compensatory beliefs model [21], when goals associated with pleasure and harm come into conflict (e.g., “this cake will be tasty but it is unhealthy”), a negative intrapersonal state of cognitive dissonance is created. Cognitive dissonance reflects an aversive motivational state that occurs when an individual holds two cognitions that are inconsistent with each other [22]. This unpleasant state can be resolved, among other ways, by altering one of the two dissonant cognitions, and it is through this pathway that compensatory beliefs are likely to operate. That is, the creation or activation of compensatory beliefs resolves cognitive dissonance because the negative health-related cognitions involved in the dissonance are likely to be reduced or be eliminated altogether (*i.e.*, consumption of the cake is no longer seen as detrimental to health because of the perceived neutralizing impact of exercise).

Conceptual work indicates that compensatory health beliefs are more likely to be active when individuals experience controlled motivation for a task [23]. As previously mentioned, compensatory beliefs become active when people face a conflict between goals to maximize pleasure and to minimize harm. In the case of controlled exercisers, the experience of physical activity satisfies the goal to minimize harm—Most individuals are likely to recognize at least some health benefits of physical exercise. However, these individuals are less likely to simultaneously satisfy their desire to experience pleasure while undertaking physical activity, so the potential for conflict between maximizing pleasure and avoiding harm is salient for these people. Goal conflict is less problematic for those who are autonomously motivated for exercise, because their experience of autonomous functioning is likely to satisfy both positive instrumental and affective goals (e.g., [24]). As such, individuals who are controlled in their exercise regulation are more likely to activate compensatory beliefs to satisfy their multiple goals, and preliminary empirical work by Miquelon, Knäuper, and Vallerand [25] has supported this premise. These authors found that autonomous motivation toward weight loss lessened participants’ activation of dietary compensatory beliefs, and that compensatory beliefs weakened consistency in adherence to dieting rules.

Fishbach and colleagues [26,27] provide more support for the notion that controlled motivation for exercise should increase post-exercise licensing for snack food consumption. Over a series of studies, Fishbach and Dhar [26] found that self-regulation through goal balancing (*i.e.*, the management of multiple and often contradictory goals) hinges on a focus on goal progress. That is, when individuals perceive that goal progress has been made on one goal (e.g., toward good health), they are likely to switch to the pursuit of alternative, often contradictory, goals (e.g., consumption of unhealthy but pleasurable food) in order to balance their multiple activated goals. This effect of goal switching has been found to be attenuated, however, when individuals focus on goal commitment rather than goal progress [26]. When goal commitment is focused upon, an action (e.g., exercise) is framed as a defining feature of one’s self-concept, and the pursuit of contradictory goals (e.g., post-exercise snacking) subsequently becomes more unappealing. This theorizing and research relates to our discussion on motivation insofar as regulations discussed in self-determination theory are defined according to their relevance to the self [28]. Autonomous motivation, by definition, is characterized by a sense of agency, volition, and identity, whereas controlled motivation is reflected in a sense of inauthenticity and pressure. In sum, autonomously motivated exercisers, as opposed to those possessing controlled motivation, are more likely to embrace a goal commitment focus during/post exercise, and this focus should dampen their desire to pursue the contradictory goal of unhealthy snack consumption.

As a final note on reflective processes, it is worth highlighting that the consumption of pleasurable high fat and/or sugary food is likely to be a common choice for consciously-mediated compensation responses post-exercise. These foods have strong effects on reward circuits in the brain involving neurotransmitters, such as dopamine, that are implicated in the experience of pleasure [29,30]. Moreover, compared to other pleasurable activities, snack food consumption is often cheap, quick, and relatively socially acceptable (as opposed to activities such as alcohol or illicit drug use). Compensatory beliefs involving exercise and diet are also likely to be activated as a function of the common perception that energy intake from foods can be expended or “neutralized” via exercise. For all these reasons, snacking and exercise are likely to be common pairings in people’s compensatory belief system. This hypothesis has been indirectly supported in recent work in which 75% of undergraduate participants reported compensatory eating post-exercise [31], although it must be noted that this statistic reflects compensatory eating rather than compensatory beliefs/justifications, Clearly though, food-related compensatory responses are common after exercise, and there is emerging theoretical and empirical evidence to suggest that these snacking-related justifications are particularly common among exercisers experiencing controlled motivation.

### 2.2. Exercise Motivation and Impulsive Post-Exercise Snack Consumption

Compensatory health beliefs develop via reflective cognitive processes, which operate (relatively) slowly by integrating knowledge about the value and probability of consequences to reach a consciously mediated decision [32]. As a result of repeated pairings between exercise and snacking behavior, however, compensatory responses may also be elicited by impulsive cognitive processes, which operate by retrieving particular associations that are activated automatically when one encounters a relevant stimulus [33]. This activation process does not require much cognitive capacity or an intention to evaluate or activate an object or idea. Instead, operations in this system occur quickly, and they are generated as a function of: (a) the preexisting structure of associations in memory; and (b) the particular set of external input stimuli. These operations can produce fast affective and nonaffective outcomes, and they can guide behavior via the activation of behavioral schemata [32]. To illustrate the mechanisms of the impulsive system, consider a situation in which an individual, after each exercise session, consciously licenses him/herself to consume a sugary drink from a vending machine. Over time, as a result of repeated pairings between exercise and sugary drink consumption, the two behaviors can become structurally associated in memory. If the individual continues to engage in the same type of exercise and in the same location, then the mere act of exercising can elicit a tendency to purchase and consume a sugary drink with little (or even without any) conscious decision process. That is, even though the drink consumption may have originally been regulated by the reflective system, over time it can become governed primarily by the impulsive system.

Even in the absence of structural associations in memory between exercise and snacking, controlled-motivated exercisers are more likely to engage in post-exercise snacking by virtue of their increased susceptibility to acting on impulse. One of the most ubiquitous contemporary models of impulsivity is that of the strength model of self-control (e.g., [19,34]), and a brief description of this model is necessary to elucidate how exercise motivation can influence impulsive snack behavior. According to proponents of the strength model, self-control is defined as the ability to override natural impulses and automatic or habitual responses [34], and it is seen as a limited intra-individual resource that can become depleted with use. In other words, individuals’ ongoing ability to exert self-control is thought to hinge on their prior usage of self-control, and when self-control strength is exhausted, a state of “ego depletion” is experienced. A large body of empirical work on this state provides general support for the strength model. That is, generally speaking, when individuals undertake a task in which self-control is needed, they often perform significantly worse in a subsequent task requiring self-control (*i.e.*, they are more likely to act on their impulses; for a review, see [35]). These failures in self-control seem to be especially likely when the self-control strategies in the second task are different to those needed for the first task [36,37], as would be the case in exercise regulation (which require approach-based strategies) and post-exercise snacking (which require avoidance-based strategies).

By definition, behaviors driven by controlled motivation require self-control. These activities are not experienced as enjoyable, fully endorsed, or valued; instead, they are associated with pressures that will encourage withdrawal from parts of the activity or from the activity altogether (see e.g., [38]). Continued pursuit of these activities will require self-control insofar as participants will need to override their desire to withdraw from the unappealing activity. Moreover, according to principles of the strength model, when individuals exert self-control in exercise, they are likely to suffer from failures of self-control in the aftermath of the exercise. This means that, irrespective of the activation of conscious compensatory beliefs, controlled exercise motivation should increase impulsive snacking post-exercise. Preliminary work by Magaraggia, Dimmock, and Jackson [39] has provided support for this hypothesis. More specifically, these authors found that students snacked on more glucose-rich food (*i.e.*, jelly beans) during a learning task that had been framed in controlling conditions rather than more autonomous conditions. Although the focus in the study by Magaraggia *et al.* [39] was on learning motivation rather than exercise motivation, there is little reason to suspect that the mechanisms associated with this effect vary across contexts. In addition, the type of snacking they observed in their study was likely to be governed by impulsive cognitive processes, since a manipulation check performed by the authors uncovered that the participants had been attentive to the learning material throughout the activity.

So far in this section, we have discussed the role of exercise motivation in impulsive snacking, but it is important to acknowledge that automatic attitudes toward food are crucial in accounting for snacking behavior via impulsive cognitive processes. Literature indicates that individuals rely more heavily on their automatic attitudes to guide behavior when ego depleted [40], so the influence of an ego depleted state on unhealthy snack consumption will be moderated by automatic attitudes toward particular unhealthy snacks. In a measure of implicit attitudes toward food, some individuals might evaluate fruit and/or vegetables more positively than high fat/sugar foods, and an ego depleted state in these individuals should not be disadvantageous from a dietary perspective—Their impulse, which has become more difficult to override, is to consume healthy rather than unhealthy foods. Ultimately, ego depletion increases our reliance on our impulses, whatever those impulses might be.

### 2.3. Exercise Motivation and Physiological Regulation of Post-Exercise Snack Behavior

Our discussion of ego depletion has focused on the effects of this state on an individual’s reliance on implicit cognitive processes in guiding behavior. Another body of literature on ego depletion is oriented toward the physiological consequences of this state. Recent theorizing and research has indicated that acts of self-control might be associated with the utilization of fuel substrates in the body (for a review, see [35]). Gailliot *et al.* [41], for example, found that participants who engaged in self-control tasks possessed lower levels of post-task blood glucose relative to a control group, and that lowered blood glucose was subsequently associated with weakened self-control. This may be of relevance given that circulating blood glucose has been implicated in the regulation of appetite for more than 50 years [42]. Research also indicates that when ego depleted, individuals consume more glucose-rich foods [39], and they may also up-regulate self-control strength by consuming them (e.g., [43]). Consequently, exercise driven by controlled motivation may encourage approach behavior toward glucose-rich foods due to physiological reasons associated with ego depletion.

In addition to the potential for ego-depletion to influence the physiological control of appetite via glucose, a relationship between motivation for exercise and the consumption of unhealthy, non-nutritious foods post-exercise may be influenced by other physiological signals of hunger. Although no previous research has investigated a role for hormonal signals in mediating a relationship between exercise motivation and post-exercise snack consumption, there is some evidence to suggest that altering one’s mindset alone can influence the circulating concentrations of appetite-regulating peptides. Crum, Corbin, Brownell, and Salovey [44] investigated the effect of influencing an individual’s perception of calorie intake on the hunger-stimulating hormone, ghrelin. In this study, participants were given an identical 380-calorie milkshake to consume on two separate occasions in a counterbalanced cross-over design; on one occasion the milkshake was presented as “indulgent” and “decadent” while on the alternate occasion it was promoted as a healthy “sensible” shake. The mindset of indulgence was associated with a steeper decline in ghrelin, compared with consumption of the identical shake when perceived as sensible. This raises the intriguing possibility that the approach to exercise, particularly in relation to motivation, may also influence physiological markers of appetite. Future studies are needed to address this issue.

A large body of literature on the physiological consequences of stress is also relevant to the proposition that motivation for exercise may influence the hormonal regulation of appetite. A potential alignment between controlled motivation and stress was highlighted in early work on self-determination theory; some 30 years ago, this category of motivation was associated with personality constructs that are maladaptive in relation to stress, such as type A personality, a sense of pressure in achievement contexts, and a sense of public self-consciousness [5]. More recently, controlled motivation has been linked to behaviors that are synonymous with stress, such as road rage, angry driving, and hostile gestures [45]. According to Weinstein and Ryan [46], four pathways exist to explain how a controlled motivation style can influence stress. First, controlled motivation evokes less processing of emotions, which over time decreases emotional health and increases stress. Second, controlled regulation encourages individuals to approach tasks defensively, anticipating them as threats rather than challenges. Third, controlled regulation encourages less interest-taking in one’s experiences, which in turn increases stress, and finally, individuals who feel controlled are likely to engage in certain behaviors, and set goals, that induce more stress.

Although one’s mindset toward stress has an effect on its consequences [47], the physiological characteristics of stress typically involve activation of the sympathetic nervous system, a parasympathetic withdrawal, and increased activity of the hypothalamic-pituitary-adrenal axis [48]. The normal hormonal pathway of the stress response leads to an increase of circulating cortisol, which promotes appetite in order to replenish fuel supplies utilised during the stress response [49]. While this is desirable in the short term, the repeated elicitation of the stress response, coupled with the ready availability of highly palatable food, leads to the preferential consumption of these foods [50,51]. Furthermore, the intake of these pleasurable but unhealthy foods tends to be self-reinforcing, because such consumption attenuates hyperactivity of the hypothalamic-pituitary-adrenal axis [52]. Of relevance, there is evidence in the literature to suggest that a controlling environment, relative to an autonomy supportive environment, increases cortisol reactivity among students [53]. There is also some limited evidence to suggest that motivation for exercise may influence circulating levels of cortisol. For instance, Di Bartolo, Lin, Montoya, Neal, and Shaffer [54] reported a significant negative correlation between salivary cortisol and exercise motivations that are synonymous with autonomous functioning. In other research, lower cortisol responses were observed in dancers who experienced greater satisfaction of basic needs that are important drivers of autonomous motivation [55]. Based on this promising preliminary evidence, further research is required to determine whether the potential influence of motivation for exercise on cortisol has implications for appetite and food intake, and whether motivation for exercise influences other hormonal regulators of appetite.

## 3. Conclusions

Physical exercise offers a number of health benefits that have been well articulated in previous work (e.g., [6]). Many of these benefits are unequivocal—There is a rich body of evidence to support them—And although there are some potential costs to exercise, such as the possibility of injury, many people see these costs as trivial when weighed against the array of desirable outcomes. Despite widespread knowledge about its health benefits, appreciation for exercise has arisen from analyses of it as an isolated category of behavior. The problem with this approach is that we can often fail to acknowledge or appreciate the possibility that exercise might influence, or be influenced by, other health behaviors. As such, the overall impact of physical exercise on health may be more complicated than previously articulated. In this article, we have argued that exercise driven by controlled motivation is likely to leave individuals susceptible to post-exercise snacking. We highlighted three possible, overlapping reasons for this effect—Via reflective cognitive processes, impulsive cognitive processes, and physiological processes—And together, these reasons could synergistically exert powerful behavioral effects on snacking.

It should be noted that we would always promote exercise as a profoundly beneficial behavior for one’s health. The arguments in this paper are not intended to attack the virtues of exercise in any way. Instead, we wish to raise the profile of compensatory exercise/food beliefs as problematic and complex cognitions. It is noteworthy that there are numerous methods that have been shown to facilitate autonomous motivation, including methods within the realm of physical exercise (e.g., [56]). We recommend that health practitioners utilize these methods to facilitate their clients’ autonomous motivation for exercise, as well as become aware of their clients’ susceptibility to engage in post-exercise snacking. We hope that our arguments in this paper stimulate research on the topic of snack responses following (and perhaps even in anticipation of) exercise. On a broader level, we hope that our work encourages researchers to consider the complex relationships between health behaviors when investigating the health-related consequences of isolated behaviors. 
